# Data on combustion, performance and emissions of a 6.8 L, 6-cylinder, Tier II diesel engine

**DOI:** 10.1016/j.dib.2020.106580

**Published:** 2020-11-26

**Authors:** Wan Nurdiyana Wan Mansor, Samsuri Abdullah, Mohammad Nor Khasbi Jarkoni, Jennifer S. Vaughn, Daniel B. Olsen

**Affiliations:** a Faculty of Ocean Engineering Technology & Informatics, Universiti Malaysia Terengganu, 21300, Kuala Nerus, Malaysia; b Fuels and Engine Research Interest Group, Universiti Malaysia Terengganu, 21300, Kuala Nerus, Malaysia; c Department of Mechanical Engineering, Colorado State University, Fort Collins, CO, 80523, United States; d Powerhouse Energy Campus, 430 N College Avenue Fort Collins, CO 80524United States

**Keywords:** Diesel engine, Tier Ii, Emissions and performance, Combustion

## Abstract

A diesel engine has been a desirable machine due to its better fuel efficiency, reliability, and higher power output. It is widely used in transportations, locomotives, power generation, and industrial applications. The combustion of diesel fuel emits harmful emissions such as unburned hydrocarbons (HC), particulate matter (PM), nitrogen oxides (NO_x_), and carbon monoxides (CO). This article presents data on the efficiency, combustion, and emission of a 4-stroke diesel engine. The engine is a 6.8 L turbocharged 6-cylinder Tier II diesel engine fitted with a common rail injection system. The test was carried out at the Powerhouse Energy Campus, Colorado State University Engines and Energy Conversion facility. The ISO Standard 8178:4 Cycle D2 cycle was adopted for this study consists of five test runs at 1800 rpm. During the testing, CO, carbon dioxide (CO_2_), oxygen (O_2_), NO_x_, PM, unburned HC as a total HC (THC), methane (CH_4_), formaldehyde (CH_2_O), and volatile organic compound (VOC) emissions were measured. At the same time, the data acquisition system recorded the combustion data. The engine's performance is characterized by the brake specific fuel combustion (BSFC) and thermal efficiency. A dataset of correlations among the parameters was also presented in this article.

**Specifications Table**

**Table:** 

Subject	Mechanical Engineering
Specific subject area	Automotive
Type of data	Table
	Figure
	Graph
How data were acquired	The performance and combustion data of the diesel were acquired using the John Deere 6068H diesel engine. The engine is a turbocharged Tier II 4-stroke direct injection engine, aftercooled with a rated power of 205 kW at 2400 rpm. The CO, CO_2_, O_2_, NO_x,_ and THC, emissions data were measured using the Rosemount 5-gas analyzer with Siemens instrument. A dilution tunnel is used for measuring PM in the exhaust. FTIR measure CH_4_, CH_2_O, and VOC.
Data format	Processed, raw
Parameters for data collection	Parameters such as pressure, heat release rate, average peak pressure, maximum pressure, indicated mean effective pressure (IMEP), BSFC, thermal efficiency, ignition delay, and 10–90% burn duration were measured and calculated. The engine was maintained constant at 1800 rpm. The emission data such as THC, CO, NO_x_, CH_4_, O_2_, CO_2_, CH_2_O, VOC, and PM were also collected. The dilution tunnel ratio of 15:1 is used
Description of data collection	Experiments were conducted under steady conditions (when the intake manifold reaches 43 °C and coolant temperature is 88 °C). Data were obtained at five engine loads corresponding to 12%, 25%, 50%, 75%, and 100% of the experiment setup's full load. The engine was stabilized for 15 min at each load, then recorded data points from emission analyzers for 10mins. Data points for combustion were recorded for 1000 cycles. During the testing, fuel flow data were also recorded for 2–3 mins.
Data source location	Powerhouse Energy Campus, 430 North College Avenue, Fort Collins, CO 80,524
Data accessibility	Data are available within this article.

**Value of the Data**

• Performance engine data is critical for ensuring the efficiency and reliability of the engine operation. It helps to conserve fuel and optimize fuel consumption.
• The data include critical details about the sum of NO_x_, HC, PM, CO and hazardous contaminants from a 6.8 L, 6-cylinder diesel engine. This data is crucial as air pollution can penetrate the bloodstream and is transmitted to human internal organs such as the lung and brain. It can result in significant health issues, including asthma, cardiovascular, cancer, decreasing the quality and number of years of life.
• Data are valuable for policymakers to further study low-emission vehicles as an attempt to combat climate change issues.
• To comply with the regulatory restrictions, the engine operator may also use the data for further engine development coupled with alternative fuel such as biodiesel or natural gas, either experimentally or numerically.

## 1 Data Description

**Fig. 1:**
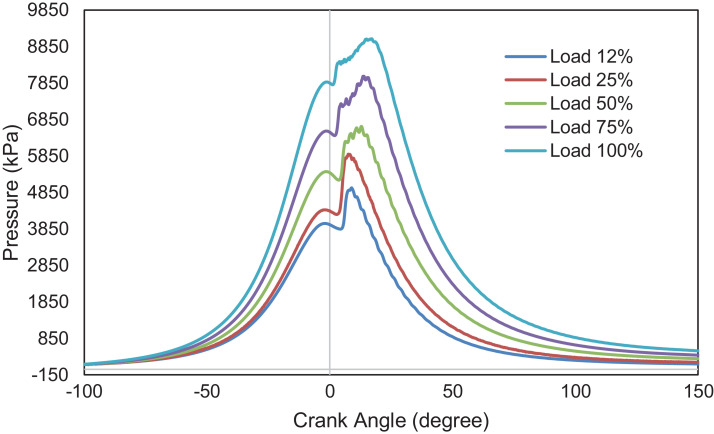
Combustion pressure profile at various loads.

**Fig. 2:**
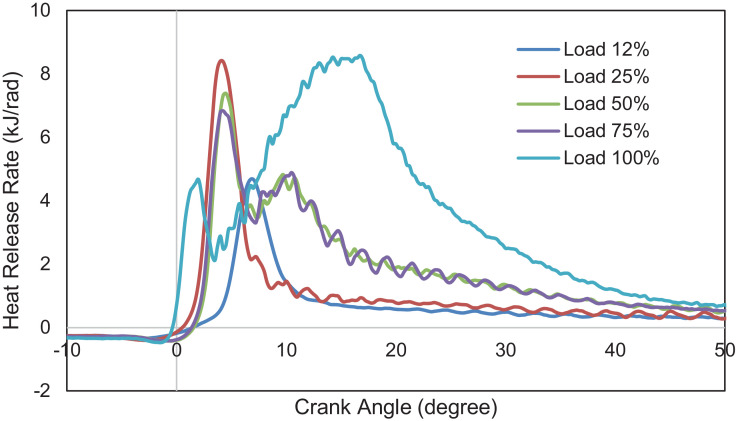
Heat release rate as a function of crank angle degree at various loads.

**Fig. 3:**
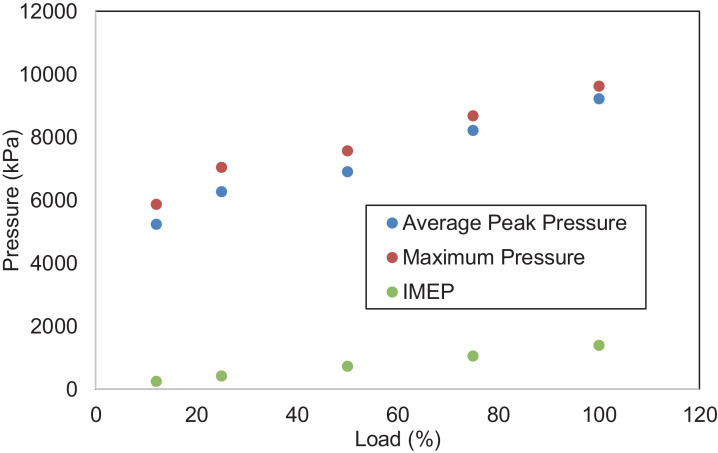
Pressure properties of a diesel engine under various loads.

**Fig. 4:**
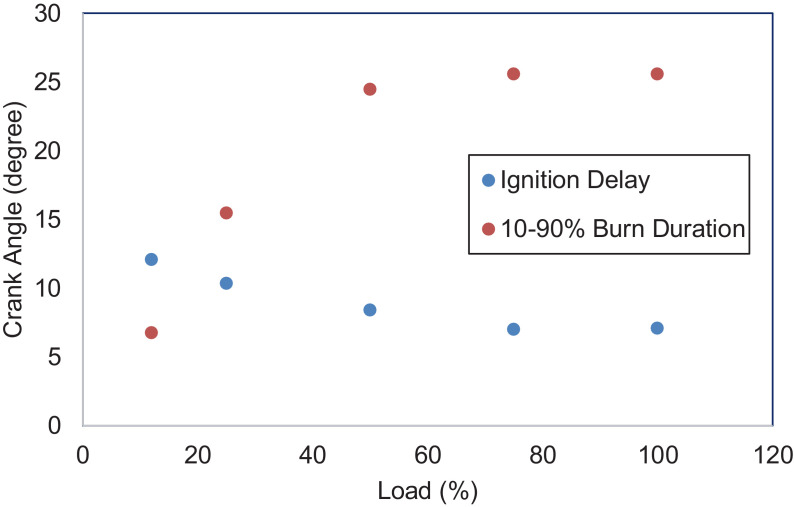
Ignition delay and burn duration of a diesel engine under various loads.

An experimental investigation was carried out using a 6.8 L, six-cylinder diesel engine. The engine is operated at a steady speed of 1800 rpm and five different loads, i.e., 12%,25%,50%,75%, and 100%. Data for combustion, performance, and emission characteristics are presented. Raw data are provided in the Supplementary Information. The pressure is determined from the six-cylinder average and obtained from −360⁰ to 359.75⁰ crank angle degrees with 0.25⁰ interval. Data for heat release rate were collected from −156⁰ to 156⁰ crank angle with 0.25⁰ intervals. Data were filtered using a 10-point moving average filter to eliminate unnecessary pulsations from the heat release curve, as defined in the following section. The in-cylinder pressure data is necessary because it measures energy conversion from chemical energy into useful mechanical energy. The heat release rate is also crucial because it represents the amount of heat produced by a burning fuel throughout the combustion process. Fig. 1, Fig. 2 depict the in-cylinder combustion pressure trace and heat release rate at various loads. The average pressure increases with loads. As the diesel engine operates at a lean air-fuel mixture ratio, lower fuel flow rates at low loads resulted in poor air and fuel mixing. Most intake air was not burned at these loads, and higher emissions were emitted due to incomplete combustion. This is supported by data on THC and CO emissions in this report. At low loads (12% and 25%), only one combustion phase occurred, i.e., the premixed phase. The combustion ended after a few crank angle degrees. The average cylinder pressure, therefore, reaches a lower value. At intermediate and higher loads, more fuel mass is injected, which leads to a greater mixture of air and fuel. The injection also starts early, providing more time for air-fuel mixing before combustion. This implies a shorter ignition delay, higher combustion pressure and lower emissions due to incomplete combustion. This is demonstrated by the presence of two phases of heat-release, i.e., premixed combustion and mixing-controlled combustion and higher average cylinder pressure. Fig. 3 presents the average peak pressure, maximum peak pressure, and the engine's IMEP at the respective load. The IMEP is defined as the indicated work during a combustion stroke, and it is independent of the engine's size. Fig. 4 indicates the ignition delay and the burn period of the combustion cycle. The length of the ignition delay is also known as the speed of the initial stage of combustion. This duration consists of physical delay and chemical retardation. The physical delay is where the air-fuel is atomized, vaporized, and mixed, while the chemical delay is due to the reactions before combustion [1]. The primary cycle of combustion is characterized by burning durations of 10–90% degree of the crank angle.

**Fig. 5:**
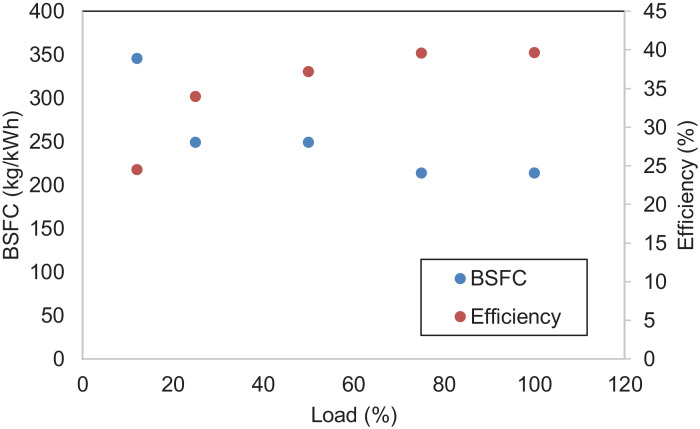
Engine's performance at five different loads.

**Fig. 6:**
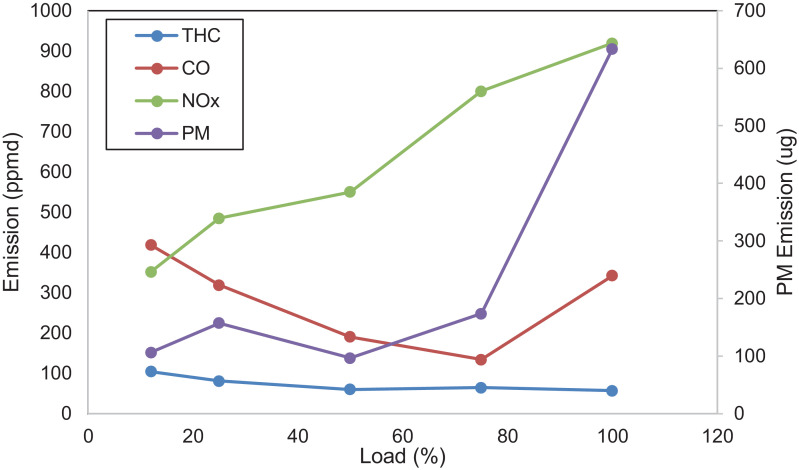
Emission concentrations of the diesel engine at five different loads.

**Fig. 7:**
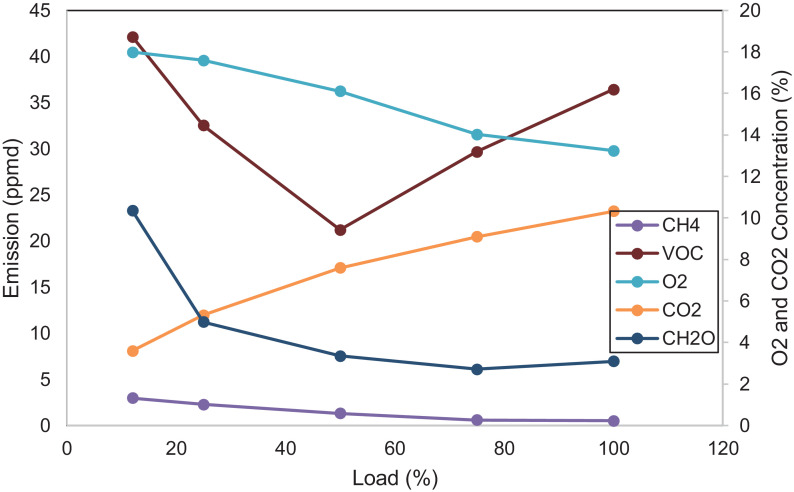
Emission concentrations of the diesel engine at five different loads.

Fig. 5 shows the performance of the diesel engine. BSFC and thermal efficiency are characteristics of the results. Fig. 6 displays the THC, CO, NO_x,_ and PM emissions of the diesel engine. NO_x_ emission is the sum of nitrogen monoxide (NO) and nitrogen dioxide (NO_2_). Fig. 7 indicates CH_4_, VOC, O_2_, CO_2,_ and CH_2_O of the diesel engine at the respective load. Table 1 indicates the Pearson correlation between the load and the combustion engine parameters. The data show a clear association between load and in-cylinder combustion pressure, i.e. average peak pressure, maximum pressure and IMEP. Data also shows a significant relationship between 10 and 90% burn duration, BSFC, and thermal efficiency. Table 2 reflects data on the correlation between load and engine emissions. Data depicts strong associations between load and NO_x_, O_2,_ and CO_2_ emissions.

**Table 1:** Pearson's correlation between load and combustion parameter.

	Load	Average Peak Pressure	Maximum Pressure	IMEP	Ignition Delay	Burn Duration	BSFC	Efficiency
Load	1							
Average Peak Pressure	.992^⁎⁎^	1						
Maximum Pressure	.987^⁎⁎^	.998^⁎⁎^	1					
IMEP	1.000^⁎⁎^	.992^⁎⁎^	.986^⁎⁎^	1				
Ignition Delay	0.190	0.193	0.152	0.203	1			
Burn Duration	0.810	0.834	0.859	0.802	−0.361	1		
BSFC	−0.796	−0.835	−0.863	−0.789	0.308	−0.991^⁎⁎^	1	
Efficiency	0.852	.883*	.905*	0.846	−0.244	.991^⁎⁎^	−0.994^⁎⁎^	1

**. Correlation is significant at the 0.01 level (2-tailed).

*. Correlation is significant at the 0.05 level (2-tailed).

**Table 2:** Pearson's correlation between load and emission parameter.

	Load	THC	CO	NO_x_	CH_4_	O_2_	CO_2_	CH_2_O	VOC	PM
Load	1									
THC	−0.855	1								
CO	−0.406	0.670	1							
NO_x_	.986^⁎⁎^	−0.817	−0.405	1						
CH_4_	−0.958*	.931*	0.650	−0.945*	1					
O_2_	−0.990^⁎⁎^	0.819	0.454	−0.986^⁎⁎^	.961^⁎⁎^	1				
CO_2_	.985^⁎⁎^	−0.927*	−0.541	.968^⁎⁎^	−0.990^⁎⁎^	−0.973^⁎⁎^	1			
CH_2_O	−0.778	.961^⁎⁎^	0.750	−0.778	.893*	0.752	−0.869	1		
VOC	−0.214	0.669	0.829	−0.155	0.450	0.191	−0.376	0.699	1	
PM	0.777	−0.488	0.242	0.773	−0.574	−0.721	0.680	−0.376	0.292	1

**. Correlation is significant at the 0.01 level (2-tailed).

*. Correlation is significant at the 0.05 level (2-tailed).

## 2 Experimental Design, Materials and Methods

**Fig. 8:**
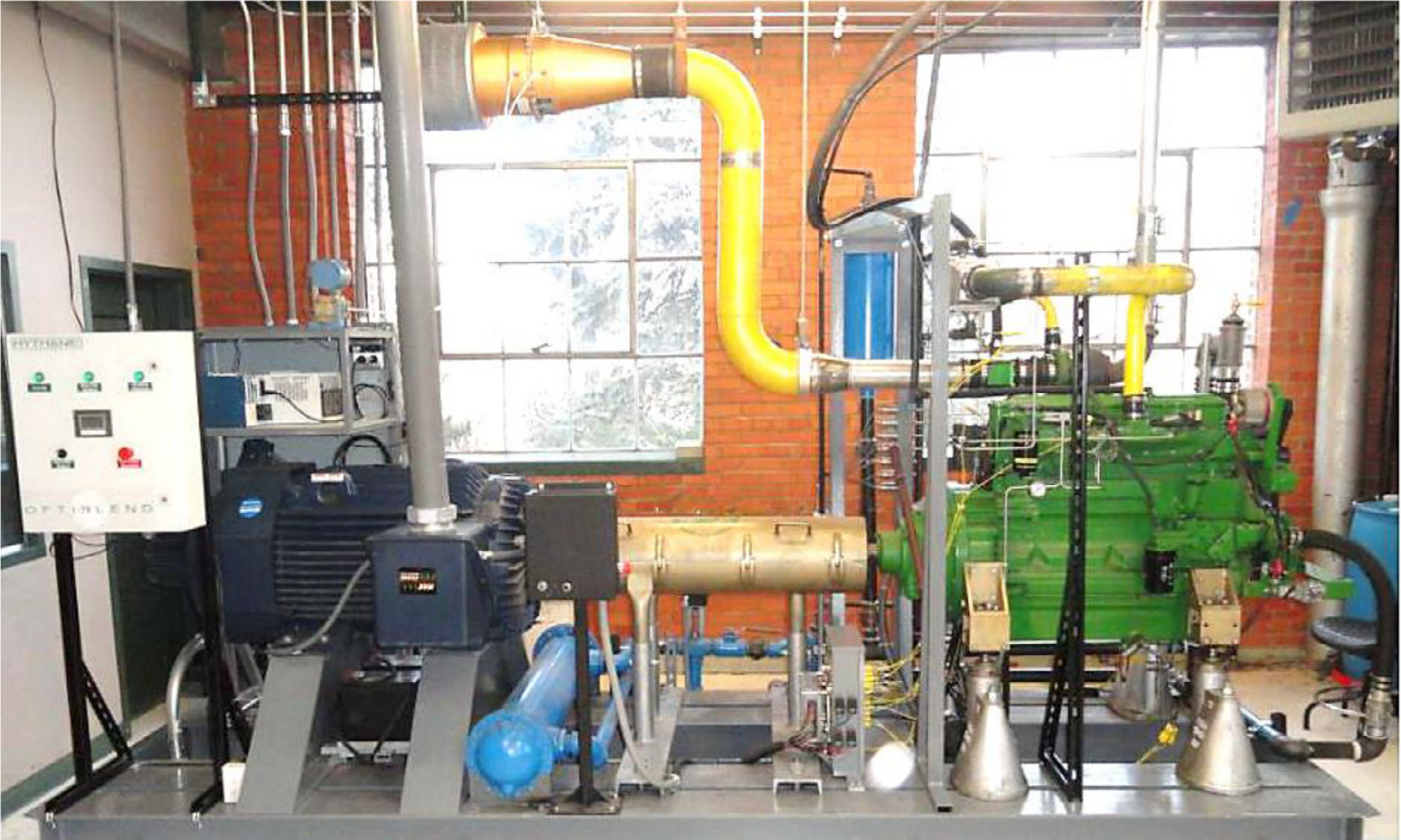
John Deere 6.8 L diesel engine setup.

Data in this article was obtained from an experimental investigation on a 4-stroke direct injection, 6.8 L, 6-cylinder John Deere diesel engine at Powerhouse Institute, Colorado State University. The engine and its basic specifications are presented in Table 3 and Fig. 8. The engine testbed is equipped with various research instruments for data collection. Kistler PiezoStar Type 6056A in-cylinder pressure sensors measure the combustion pressure data fitted with a glow plug adaptor. Type K thermocouples measure intake, exhaust, and gas temperature. The engine is also mounted with motor, torque, and engine speed sensors. The diesel fuel flow rate is measured by DevX software supplied by the engine manufacturer. An AC motor dynamometer powers the engine with a rated power of 350 hp at 480 VAC. A LabView Virtual Instrument VI is installed to monitor the device remotely.

**Table 3:** John Deere diesel engine specification.

Engine model and type	6068HF475 and In-line, 4-cycle
Number of Cylinders	6
Bore and Stroke	106 × 127 mm
Connecting Rod	203 mm
Compression Ratio	17:1
Aspiration	Turbocharged and Aftercooled
Displacement	6.8 liters
Rated Power	205 kW (275 hp)
Rated Speed	2400 rpm
Normal operation speed	1800 rpm

**Fig. 9:**
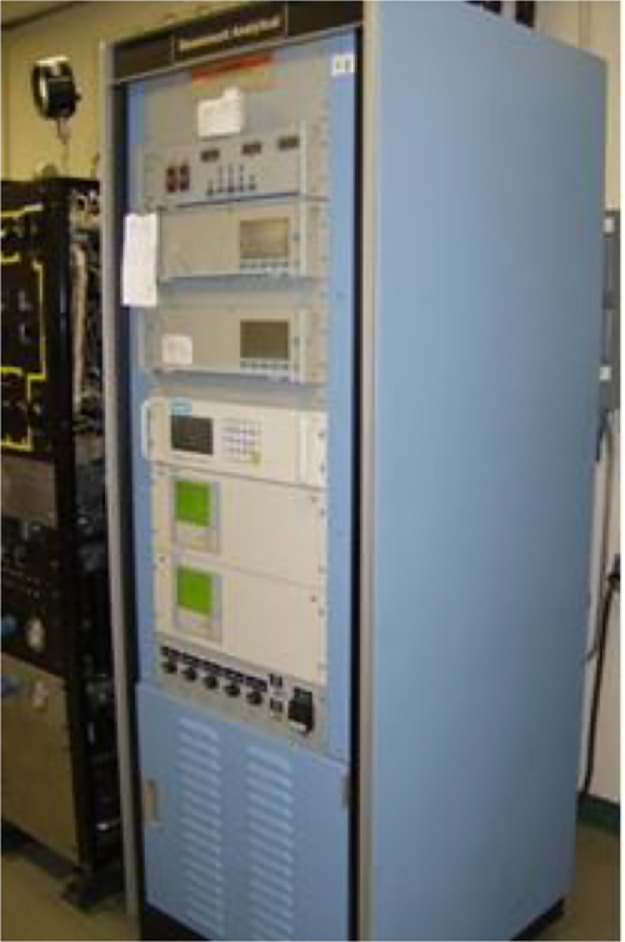
The Rosemount 5-gas analyzer with Siemens sensors.

**Fig. 10:**
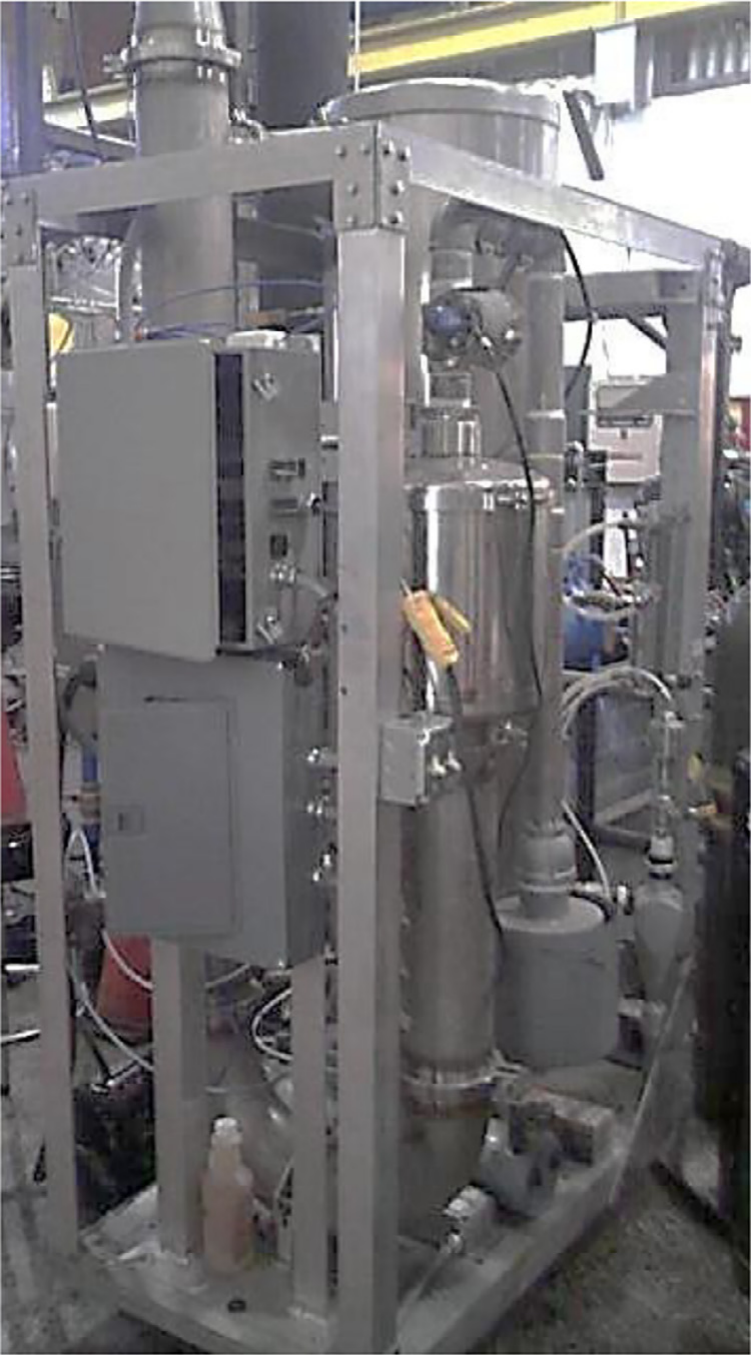
PM dilution tunnel.

**Fig. 11:**
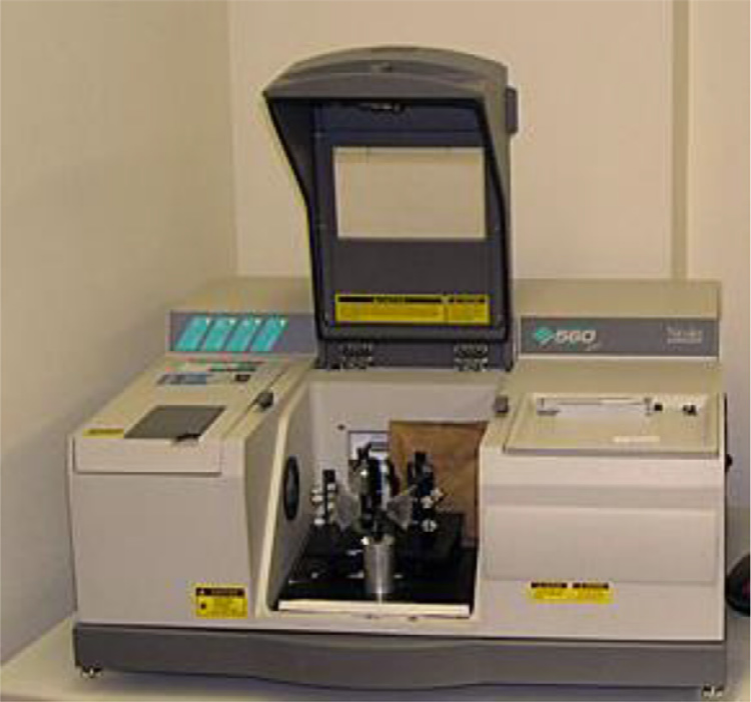
Nicolet Magna 560 FTIR spectrometer.

The Rosemount 5-gas analyzer with Siemens sensors measures CO, CO_2_, O_2_, NO_x_, and THC concentrations while a dilution tunnel measures PM with a dilution ratio of 15:1. Fig. 9, Fig. 10 show the 5-gas analyzer and dilution tunnel, respectively. The measurement method for CH_2_O, CH_4,_ and VOC concentration uses the Fourier Transform Infra-Red Spectrometer (FTIR). The Nicolet Magna 560 FTIR spectrometer used in this data collection is shown in Fig. 11. The engine is tested at a constant speed of 1800 rpm at the steady-state condition for five loads, i.e., 12%, 25%, 50%, 75%, and 100% load. Pressure data was taken three times to ensure precision and was averaged. The heat release rate is calculated using Eq. (1). This empirical method is adapted by Gatowski et al., where the combustion chamber is assumed to be a closed system in which only fuel flow is considered [2].

(1) $$\frac{dQ}{d\theta }\;\;=\;\;\left(\frac{C_{P}P}{R}\right)\frac{dV}{d\theta }\;\;+\;\;\left(\frac{C_{V}V}{R}\right)\frac{dP}{d\theta }\;\;-\;\;\frac{dQ_{w}}{d\theta }$$

where $\frac{dQ}{d\theta }$ is the transfer rate of heat as a function of the crank angle degree, $C_{P}$ and $C_{V}$ are the gas specific heats at constant pressure and volume, R is the specific ideal gas constant, $\frac{dV}{d\theta }\;$is the volume displaced over the system, $\frac{dP}{d\theta }$ is the differential pressure and $\frac{dQ_{w}}{d\theta }$ is the wall heat transfer rate.

A weighted 10-point moving average filter is used to counter the high amplitude of heat release rate data using Eq. (2) [3]. Ignition delay and burn duration are calculated using Eq. (3) and Eq. (4) [4]. Data for the start of injection timing and the mass fuel flow rate for each load are shown in Table 4. The BSFC is calculated by dividing the amount of diesel mass flow by the engine power, as shown in Eq. (5). The thermal efficiency is determined using the inversion of BSFC and the lower heating value (LHV) of the diesel, as shown in Eq. (6) [5]. Pearson's correlation analysis was processed using IBM SPSS Statistic Version 25. The SPSS represents statistical validity on the thresholds alpha = 0.05 and alpha = 0.01 [6]. The engine's operating modes and the weighting factors for cycle D2 by the ISO 8178:4 procedure for non-road engine applications are used. This cycle is shown in Table 5.

(2) $$y\left(n\right)=\frac{1}{N}\sum_{i=0}^{N-1}x\left[n-i\right]$$

where y(n) is the output signal and x(n) is the sample input.

(3) $$Ignition\;delay\left({}^{\circ }\right)=start\;of\;injection\;+\;mass\;fraction\;burned\;at\;10\%\;crank\;angle$$

(4) $$10-90\%\;burn\;duration\;\left({}^{\circ }\right)=90\%\;mass\;fraction\;burned\;--10\%\;mass\;fraction\;burned$$

(5) $$BSFC\;\;=\;\;\frac{{\overset{\cdot }{m}}_{f}}{\dot{W}}$$

(6) $$Efficiency\;=\;\frac{1}{BSFC\times LHV}$$

**Table 4:** Start of injection timing and mass fuel flow rate.

Load	12%	25%	50%	75%	100%
Start of Injection (⁰bTDC)	6.50	6.00	3.27	3.08	2.78
Mass fuel flow rate (kg/h)	5.65	8.97	15.41	21.91	29.24

**Table 5:** Weighting factors of ISO 8178 type D2 test cycles.

Load	12%	25%	50%	75%	100%
Weighing factor	0.1	0.3	0.3	0.25	0.05

## CRediT Author Statement

**Wan Nurdiyana Wan Mansor:** Conceptualization, Methodology, Formal Analysis, Writing – Original Draft, Editing. **Samsuri Abdullah:** Validation, Formal Analysis. **Mohammad Nor Khasbi Jarkoni:** Statistical Analysis, Writing. **Jennifer S. Vaughn:** Data collection, Resources, Investigation. **Daniel B. Olsen:** Conceptualization, Resources, Investigation.

## Declaration of Competing Interest

The authors have no conflict of interest to declare.

## Appendix Supplementary materials

Image, application 1

Image, application 2
